# Corrections to: Mapping spatial frequency preferences across human primary visual cortex

**DOI:** 10.1167/jov.24.13.8

**Published:** 2024-12-09

***CORRECTIONS TO:*** Broderick, W. F., Simoncelli, E. P., & Winawer, J. (2022). Mapping spatial frequency preferences across human primary visual cortex. *Journal of Vision, 22*(4), 3, https://doi.org/10.1167/jov.22.4.3.

The authors found a bug in their code where polar angle variables (retinotopic angle, stimulus orientation) were created so that the angle increased clockwise, instead of counterclockwise, which is the more standard convention. This is equivalent to a vertical flip.

Correcting the bug has no effect on the results since the unusual convention was used consistently. However, all stimuli described as having a positive ω_*a*_ actually have a negative ω_*a*_. Thus, one paragraph in the methods and one figure have been updated.

Original text of the second-to-last paragraph of the Methods/Stimulus design section:

We generated stimuli corresponding to 48 different frequency vectors (see Fig. 2), at 8 different phases ϕ ∈ {0, π/4, π/2, …, 7π/4}. The frequency vectors were organized into five different categories:

(1) Pinwheels: ω_*r*_ = 0, ω_*a*_ ∈ {6, 8, 11, 16, 23, 32, 45, 64, 91, 128}
(2) Annuli: ω_*a*_ = 0, ω_*r*_ ∈ {6, 8, 11, 16, 23, 32, 45, 64, 91, 128}
(3) Forward spirals: ω_*r*_ = ω_*a*_ ∈ {4, 6, 8, 11, 16, 23, 32, 45, 64, 91}
(4) Reverse spirals: ω_*r*_ = −ω_*a*_ ∈ {4, 6, 8, 11, 16, 23, 32, 45, 64, 91}
(5) Fixed-frequency mixtures: (ω_*r*_, ω_*a*_) ∈ {(8, 31), (16, 28), (28, 16), (31, 8), (31, −8), (28, −16), (16, −28), (8, −31)}

Corrected text:

We generated stimuli corresponding to 48 different frequency vectors (see Fig. 2), at 8 different phases ϕ ∈ {0, π/4, π/2, …, 7π/4}. The frequency vectors were organized into five different categories:

(1) Pinwheels: ω_*r*_ = 0, ω_*a*_ ∈ { − 6, −8, −11, −16, −23, −32, −45, −64, −91, −128}
(2) Annuli: ω_*a*_ = 0, ω_*r*_ ∈ {6, 8, 11, 16, 23, 32, 45, 64, 91, 128}
(3) Forward spirals: ω_*r*_ = −ω_*a*_ ∈ {4, 6, 8, 11, 16, 23, 32, 45, 64, 91}
(4) Reverse spirals: ω_*r*_ = ω_*a*_ ∈ {4, 6, 8, 11, 16, 23, 32, 45, 64, 91}
(5) Fixed-frequency mixtures: (ω_*r*_, ω_*a*_) ∈ {(8, 31), (16, 28), (28, 16), (31, 8), (31, −8), (28, −16), (16, −28), (8, −31)}

Original Figure 2 and legend:

**Figure 2.**
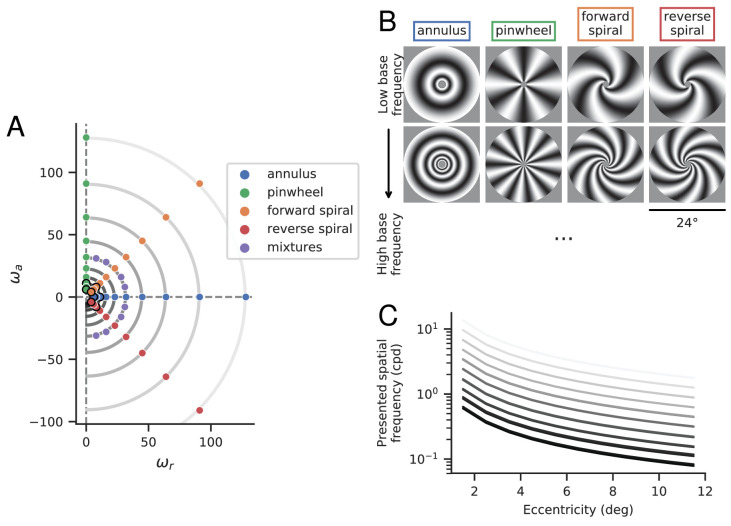
Stimuli. (**A**) Base frequencies (ω_*r*_, ω_*a*_) of experimental stimuli. Stimulus category is determined by the relationship between ω_*a*_ and ω_*r*_, which determines local orientation information (Eq. 3). (**B**) Example stimuli from four primary classes, at two different base frequencies. These stimuli correspond to the dots outlined in black in panel A. (**C**) Local spatial frequencies (in cycles per degree) as a function of eccentricity. Each curve represents stimuli with a specific base frequency, $\sqrt{\omega_{r}^{2}+\omega_{a}^{2}}$, corresponding to one of the semi-circular contours in panel A. The two rows of stimuli in panel B correspond to the bottom and 3rd-from-bottom curves.

Corrected Figure 2 and (unchanged) legend:

**Figure 2.**
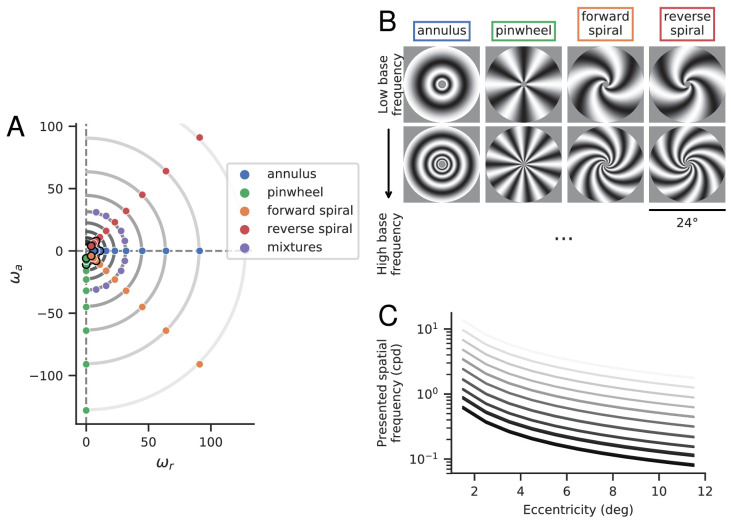
Stimuli. (**A**) Base frequencies (ω_*r*_, ω_*a*_) of experimental stimuli. Stimulus category is determined by the relationship between ω_*a*_ and ω_*r*_, which determines local orientation information (Eq. 3). (**B**) Example stimuli from four primary classes, at two different base frequencies. These stimuli correspond to the dots outlined in black in panel A. (**C**) Local spatial frequencies (in cycles per degree) as a function of eccentricity. Each curve represents stimuli with a specific base frequency, $\sqrt{\omega_{r}^{2}+\omega_{a}^{2}}$, corresponding to one of the semi-circular contours in panel A. The two rows of stimuli in panel B correspond to the bottom and 3rd-from-bottom curves.

The authors would like to thank Jiyeong Ha for discovering the original bug and alerting them to its presence.

