# Supplementary material for: Mapping spatial frequency preferences across human primary visual cortex
Source: J Vis. 2022 Mar 10;22(4):3. doi: 10.1167/jov.22.4.3 (PMC8934567; doi:10.1167/jov.22.4.3)
Supplement: Supplement 1 [file jovi-22-4-3_s001.pdf]

Supplement for "Mapping Spatial Frequency Preferences Across Human Primary Visual Cortex"

A Preprint

February 3, 2022

1 Appendix

1.1 Stimulus properties

The local spatial frequency of our stimuli is equal to the magnitude of the gradient of the argument of $\cos(\cdot)$ in Equation 1. Writing that argument as $g(r, \theta) = \omega_r \ln(r) + \omega_a \theta + \phi$, we differentiate to obtain the horizontal/vertical spatial frequency:

$$\omega_x = \frac{\partial g}{\partial x} = \frac{\partial g}{\partial r}\frac{\partial r}{\partial x} + \frac{\partial g}{\partial \theta}\frac{\partial \theta}{\partial x} = \frac{x\omega_r - y\omega_a}{x^2 + y^2} \tag{1}$$

$$\omega_y = \frac{\partial g}{\partial y} = \frac{\partial g}{\partial r}\frac{\partial r}{\partial y} + \frac{\partial g}{\partial \theta}\frac{\partial \theta}{\partial y} = \frac{y\omega_r + x\omega_a}{x^2 + y^2}. \tag{2}$$

The local spatial frequency is then

$$\begin{aligned} \omega_l &= \sqrt{\omega_x^2 + \omega_y^2} \\ &= \frac{\sqrt{(x^2 + y^2)(\omega_r^2 + \omega_a^2)}}{x^2 + y^2} \\ &= \frac{\sqrt{\omega_r^2 + \omega_a^2}}{r}. \end{aligned} \tag{3}$$

Thus, the local spatial frequency is proportional to the magnitude of the base frequency vector (ω_a, ω_r), decreasing as the inverse of eccentricity (r). To convert this from radians per pixel to cycles per degree, we multiply by a conversion factor, $c = \frac{1}{2\pi}\frac{\text{size}_{\text{pix}}}{\text{size}_{\text{deg}}}$. We use this measure of local spatial frequency when fitting tuning curves.

We can similarly find the local stimulus orientation, θ_l, by computing the angle of the frequency vector (ω_x, ω_y):

$$\begin{aligned} \theta_l &= \arctan \frac{\omega_y}{\omega_x} \\ &= \arctan \frac{y\omega_r + x\omega_a}{x\omega_r - y\omega_a} \\ &= \theta + \arctan \frac{\omega_a}{\omega_r} \end{aligned} \tag{4}$$

where $\theta = \arctan \frac{y}{x}$. The local stimulus orientation is thus the sum of the angular location θ and the angle of the base frequency vector (ω_r, ω_a).

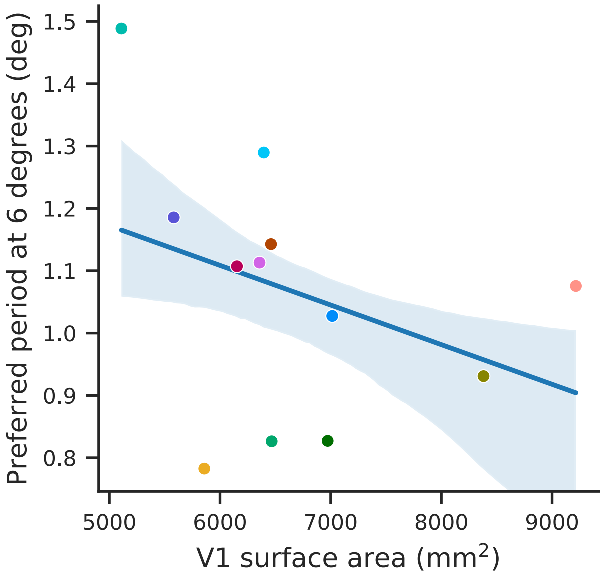

Figure S1: Scatter plot showing the preferred period at 6 degrees eccentricity against the total V1 surface area (both hemispheres) for all subjects. A middling eccentricity value was chosen so that the effects of both parameters a and b are visible. The two variables are essentially uncorrelated (line shows the median and the shaded region the 68% CI linear regression, bootstrapped across subjects). Subject colors are as in figure 9B.

1.2 Behavior

Behavioral data is plotted in figures S3 and S4, combining across subjects and plotting them separately, respectively. When combining across subjects, there is no consistent pattern between performance and stimulus type: all stimulus types show similar behavior. When looking on a subject-by-subject basis, about half of the subjects show some differences across stimulus types. We might then worry that differences in eye movements or fixation stability may affect our results, such that any differences between spatial frequency tuning for annuli and pinwheels, say, are actually the result in differences in eye movements between those conditions. However, there appears to be no relationship between these behavioral patterns and the parameter values plotted in figure 9B. For example, sub-01 and sub-08 show similar behavioral patterns, with the highest miss rates for pinwheels, followed by forward spirals, then annuli and reverse spirals. However, their parameter values are not more similar to each other than to any other subject's, making it unlikely that our parameter fits reflect differences in eye movement across stimuli.

Similarly, one might worry that differences in stimulus-independent eye movements might affect our results: fixational eye movements are known to be more common along the horizontal than vertical meridian, occuring at a rate of about 6 per minute (Thaler, Schütz, Goodale, and Gegenfurtner, 2013), with microsaccades in both directions having a median amplitude of 20 arcmin when there is a fixation target (Cherici, Kuang, Poletti, and Rucci, 2012). These increase the uncertainty of the spatial frequency within a voxel's pRF, but, whenever voxels whose pRFs are on the right horizontal meridian see an increase in eccentricity due to a horizontal eye movement, voxels on the left horizontal meridian will see a decrease in eccentricity (and vice versa). Since our model does not allow left/right asymmetries in the fits, these two effects would approximately cancel. Moreover, even if they did not fully cancel, microsaccades are relatively small compared to the observed effects of orientation (equivalent to about a 2 deg shift in eccentricity).

In addition to the uncertainty in voxel location discussed above, microsaccades combined with temporal averaging may blur the stimulus slightly, suppressing the high frequencies in our stimuli, which may shift our measured tuning curves to slightly lower frequencies. This effect would be most pronounced for those stimuli whose period is the same magnitude as the eye movements, which are present in our stimuli. This would increase the preferred periods on our plots and would have a larger effect on voxels at lower eccentricities. Eliminating or fully accounting for this effect is impossible given our setup, and future studies are necessary to account for its magnitude. Given the small size of fixational eye movements, we think their effects are likely to be small, but we cannot entirely rule them out.

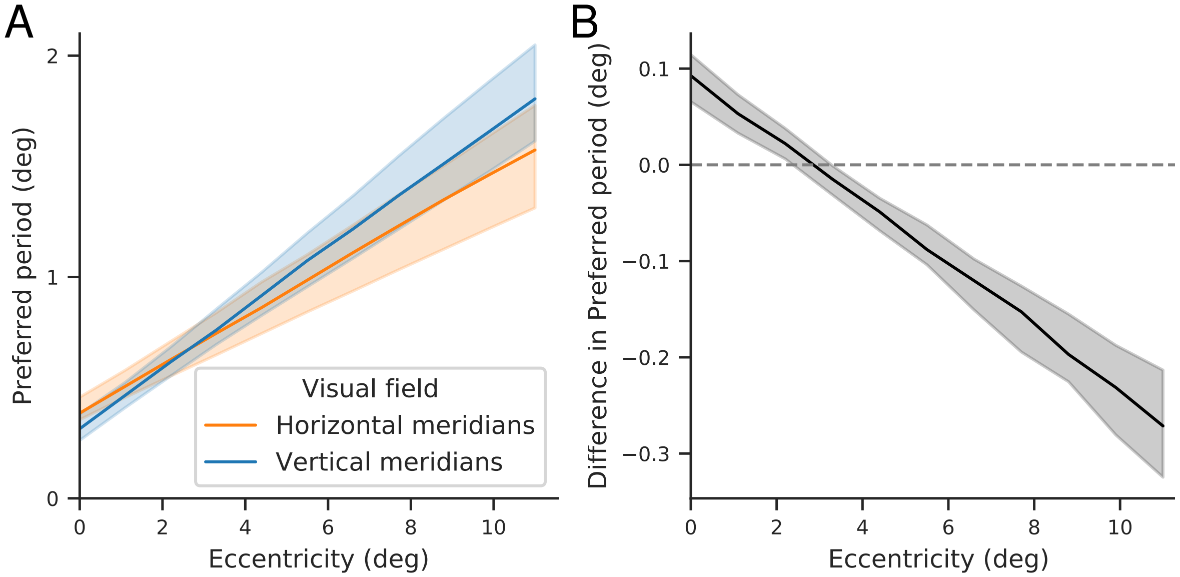

Figure S2: Voxels near the vertical meridians have a higher preferred period in the periphery and a lower preferred period near the fovea. Model 3 ($p = ar_v + b$, no effect of stimulus orientation or modulation of gain) was fit to each subject's median estimates of BOLD response. (A) Preferred period as a function of eccentricity for the two portions of the visual field. Lines and shaded region show the median and 68% CI from combining the preferred period across subjects by bootstrapping a precision-weighted average; uncertainty thus reflects both between-subject variance of preferred period and within-subject variance of the two visual field segments. (B) Difference between the preferred period in the voxels near the horizontal and vertical meridians, calculated within subjects, and then combined by bootstrapping a precision-weighted average.

1.3 Individual fits

Figures S5 through S11 show the individual subject fits for preferred period as a function of eccentricity from the 1d analysis (figure S5); preferred period as a function of eccentricity from the 2d model for relative (figure S6) and absolute (figure S7) reference frames; preferred period as a function of retinotopic angle (at 5 degrees eccentricity) for relative (figure S8) and absolute (figure S9) reference frames; and the relative gain as a function of retinotopic angle for relative (figure S10) and absolute (figure S11) reference frames. In all, we show the median and 68% confidence intervals obtained from bootstrapping across that subject's fMRI runs.

Note that sub-12's results are an outlier: their preferred period does not change as a function of eccentricity (also visible in the parameter plots in figure 9B; their $a = 0$). The noise in their GLMdenoise fits does not suggest any problems with the quality of this data, and the quality of their retinopic maps is also consistent with the other subjects. Therefore, they have been included in the analyses presented in this paper.

References

Cherici, C., Kuang, X., Poletti, M., & Rucci, M. (2012). Precision of sustained fixation in trained and untrained observers. *Journal of Vision*, *12*(6), 31–31. doi:10.1167/12.6.31

Thaler, L., Schütz, A., Goodale, M., & Gegenfurtner, K. (2013). What is the best fixation target? the effect of target shape on stability of fixational eye movements. *Vision Research*, *76*, 31–42. doi:10.1016/j.visres.2012.10.012

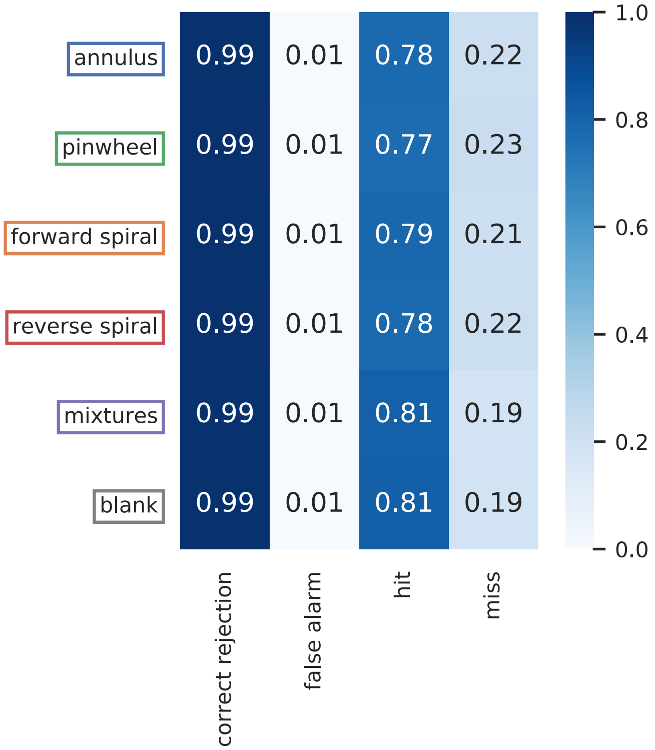

Figure S3: Summary of behavior combined across subjects. Stimulus type is indicated along the vertical axis ("blank" means there was no stimulus on the screen; these trials were interleaved throughout the scan as well as present at the very beginning and end), and outcome is indicated on the horizontal axis, with numbers giving the percentage of trials that fall into that category. Percentages and color are normalized so that the sum of correct rejections and false alarms is 1, as is the sum of hits and misses. During scans, subjects viewed a pseudo-random stream of digits at fixation and their task was to press a button whenever the digit repeated, which it did on one-sixth of the trials (the same digit was never shown three trials in a row). Behavior was consistent across stimulus types.

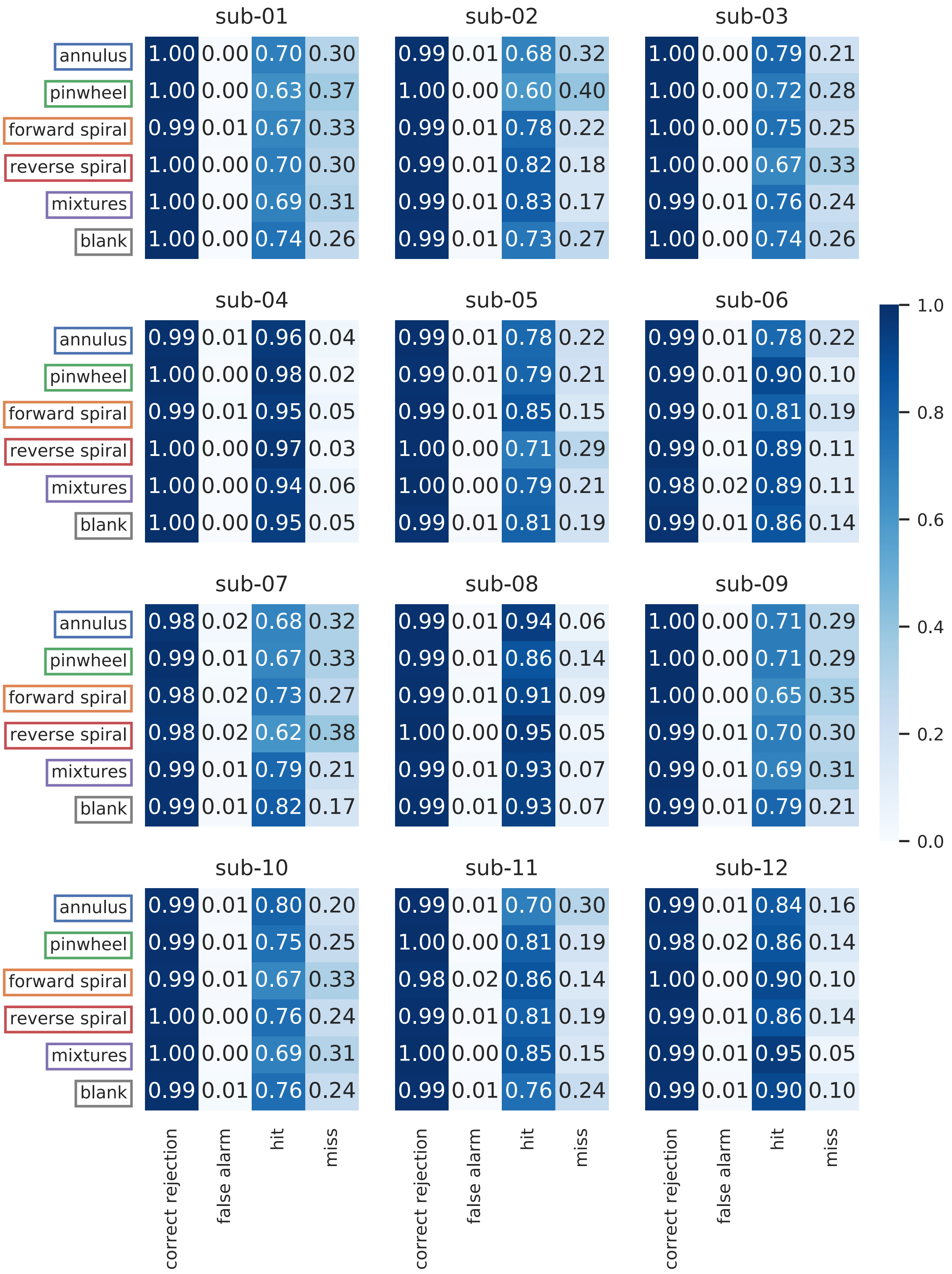

Figure S4: Summary of behavior on a per-subject basis. For details, see caption of S3. Performance varies across subjects (though false alarm rates are consistently low), but as in S3, there is no consistent difference in behavior across stimulus types.

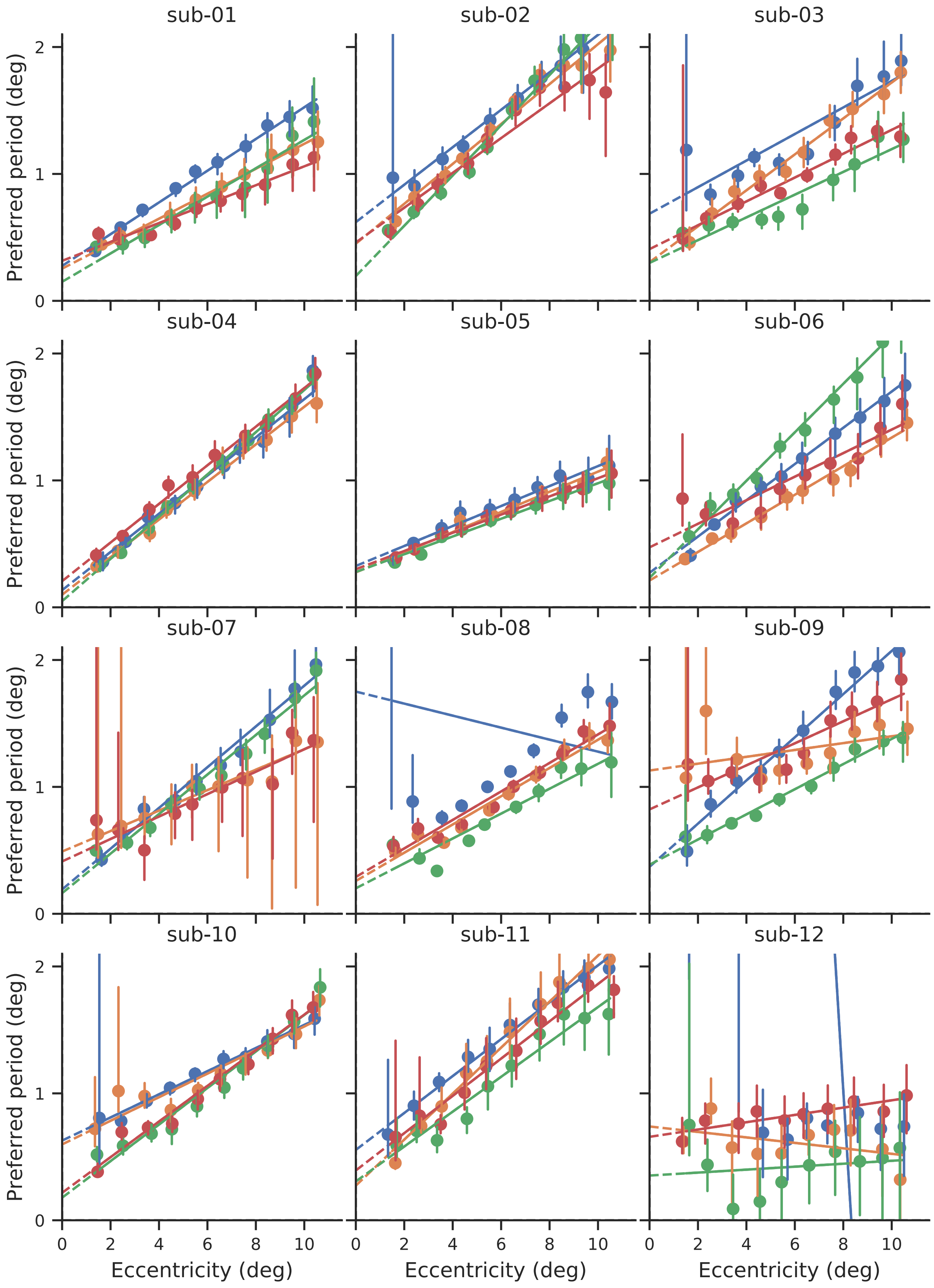

Figure S5: Individual subjects' preferred period as function of eccentricity from 1d fits (as in figure 6A), for different stimulus classes. Points and vertical bars indicate the median and 68% confidence interval obtained from bootstrapping across fMRI runs.

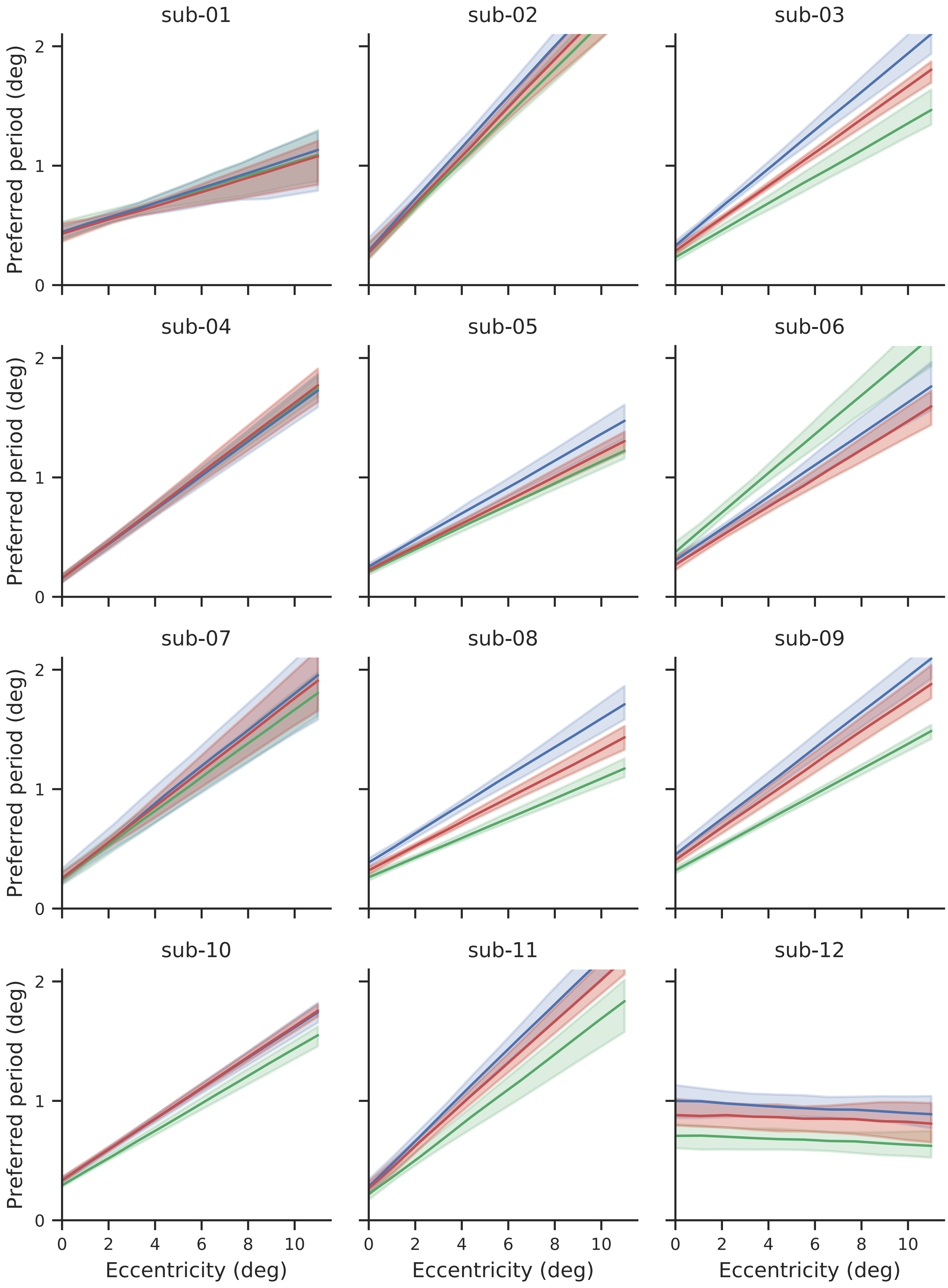

Figure S6: Individual subjects' referred period as function of eccentricity from 2d model for relative reference frame (as in left panel of figure 10A). Averaged across all angles, lines show the median parameter and shaded regions cover the 68% confidence intervals obtained from bootstrapping across fMRI runs.

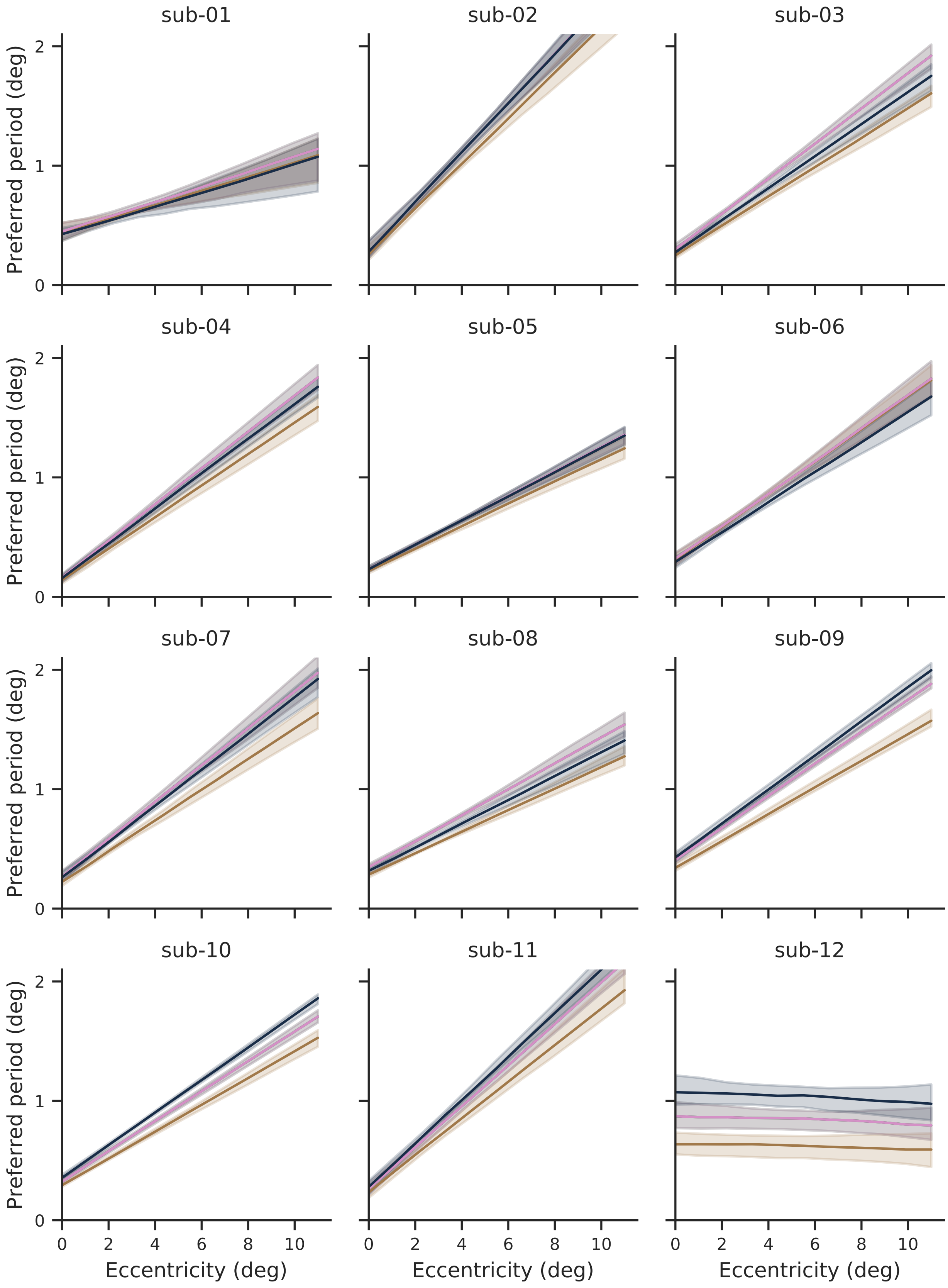

Figure S7: Individual subjects' preferred period as function of eccentricity from 2d model for absolute reference frame (as in left panel of figure 10B). Averaged across all angles, lines show the median parameter and shaded regions cover the 68% confidence intervals obtained from bootstrapping across fMRI runs.

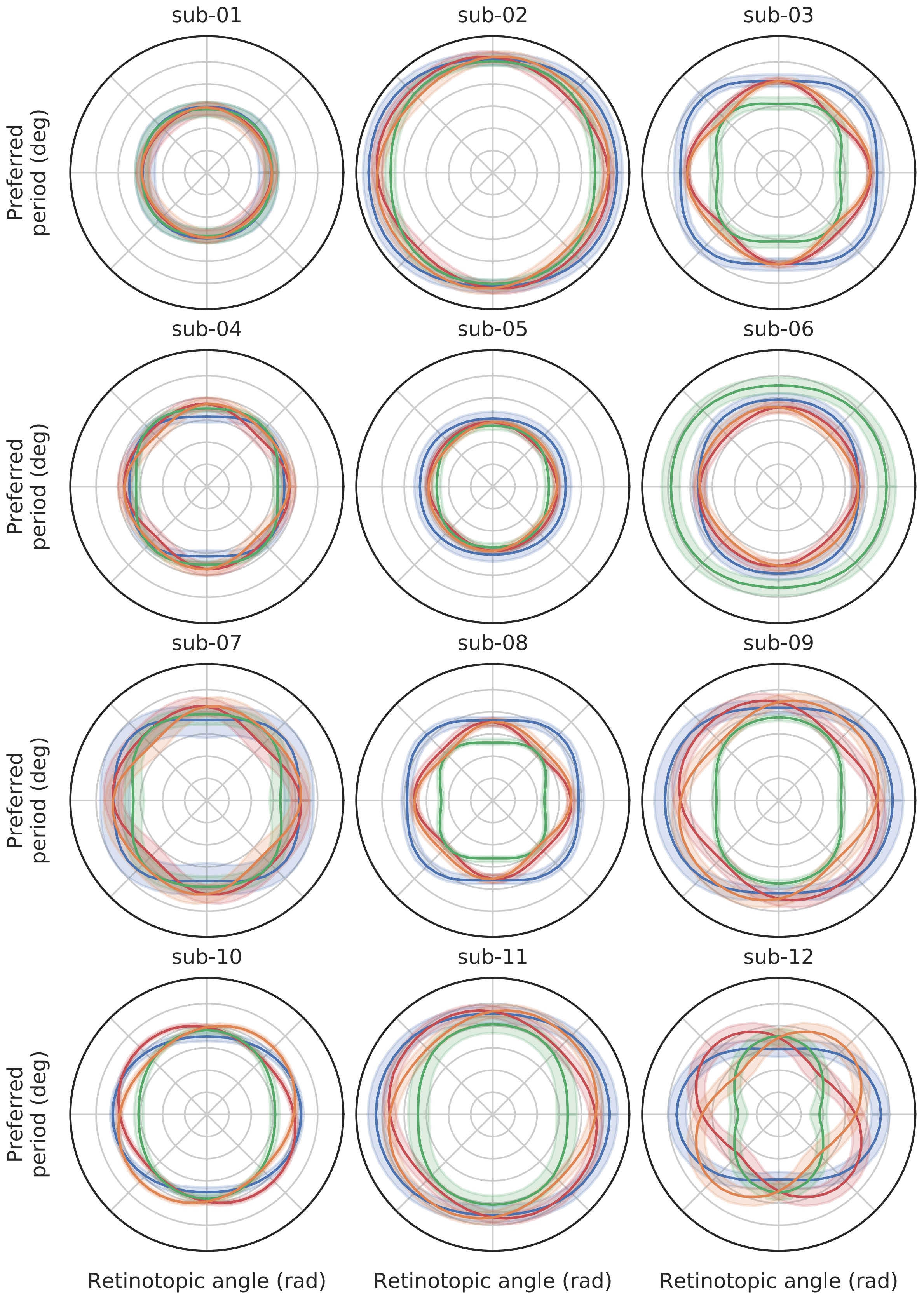

Figure S8: Individual subjects' preferred period as a function of retinotopic angle at an eccentricity of 5 degrees for relative reference frame (as in top right panel of figure 10A). Lines show the median parameter and shaded regions cover the 68% confidence intervals obtained from bootstrapping across fMRI runs.

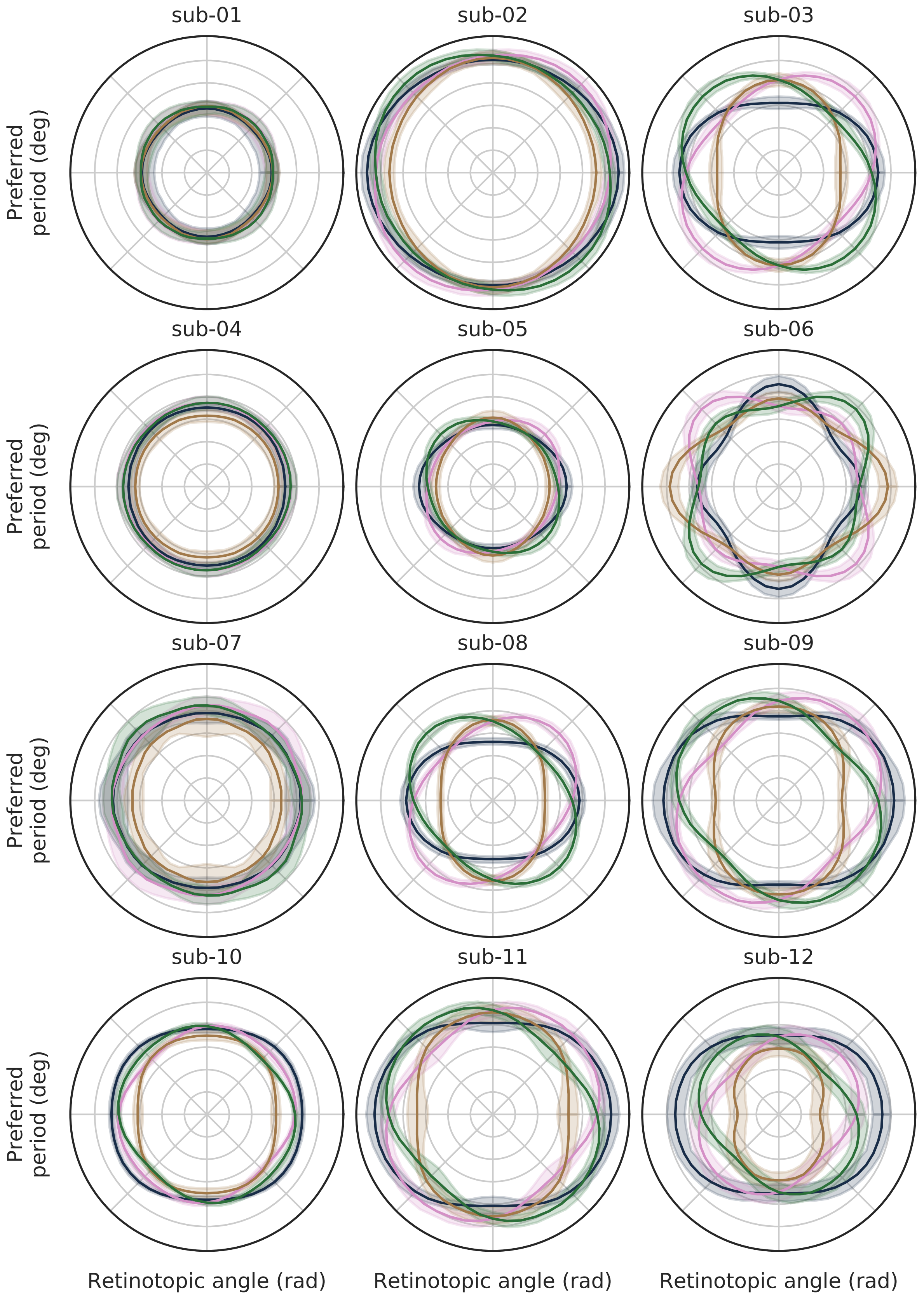

Figure S9: Individual subjects' preferred period as a function of retinotopic angle at an eccentricity of 5 degrees for absolute reference frame (as in top right panel of figure 10B). Lines show the median parameter and shaded regions cover the 68% confidence intervals obtained from bootstrapping across fMRI runs.

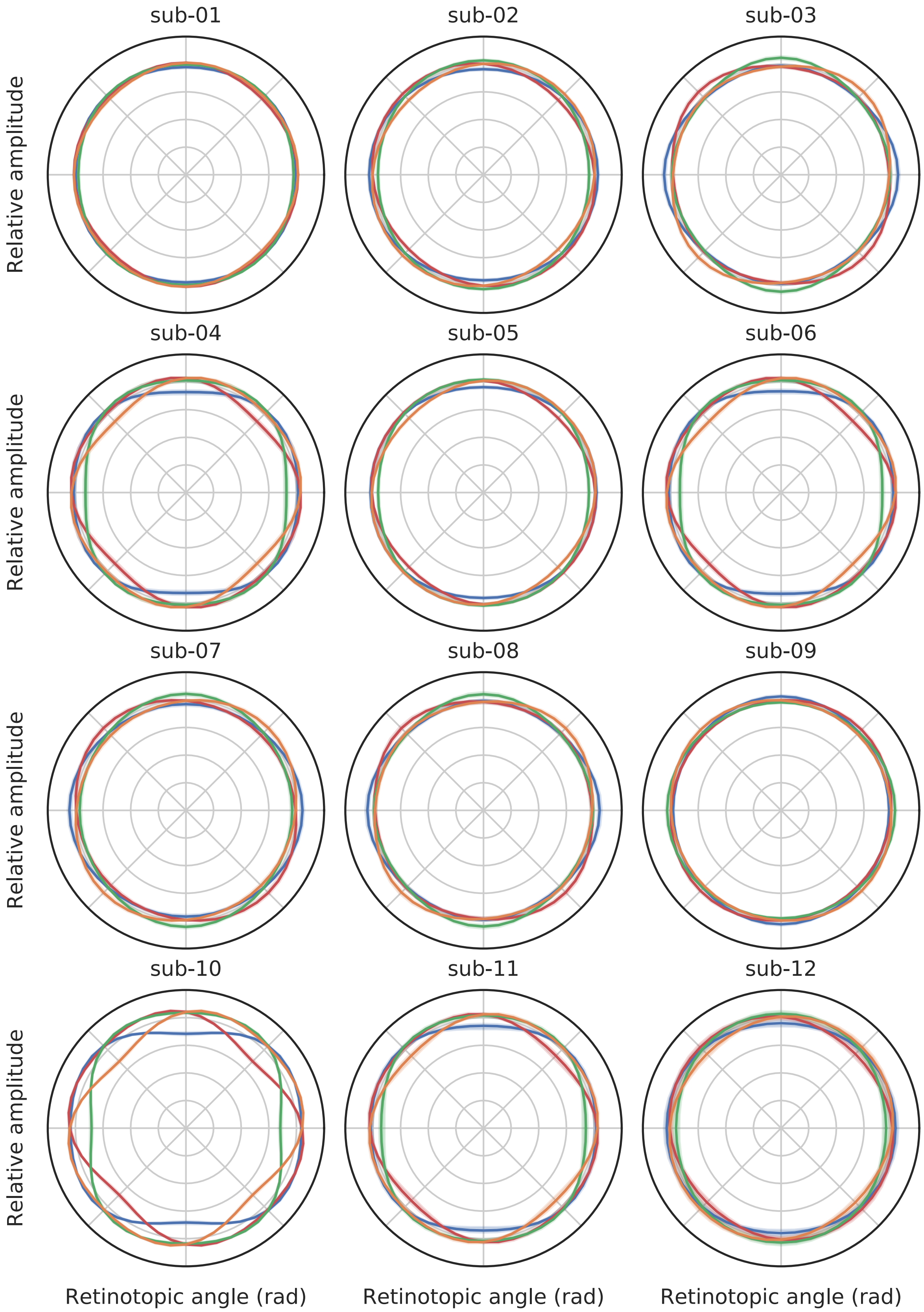

Figure S10: Individual subjects' relative gain as a function of retinotopic angle for relative reference frame (as in bottom right panel of figure 10A). Lines show the median parameter and shaded regions cover the 68% confidence intervals obtained from bootstrapping across fMRI runs.

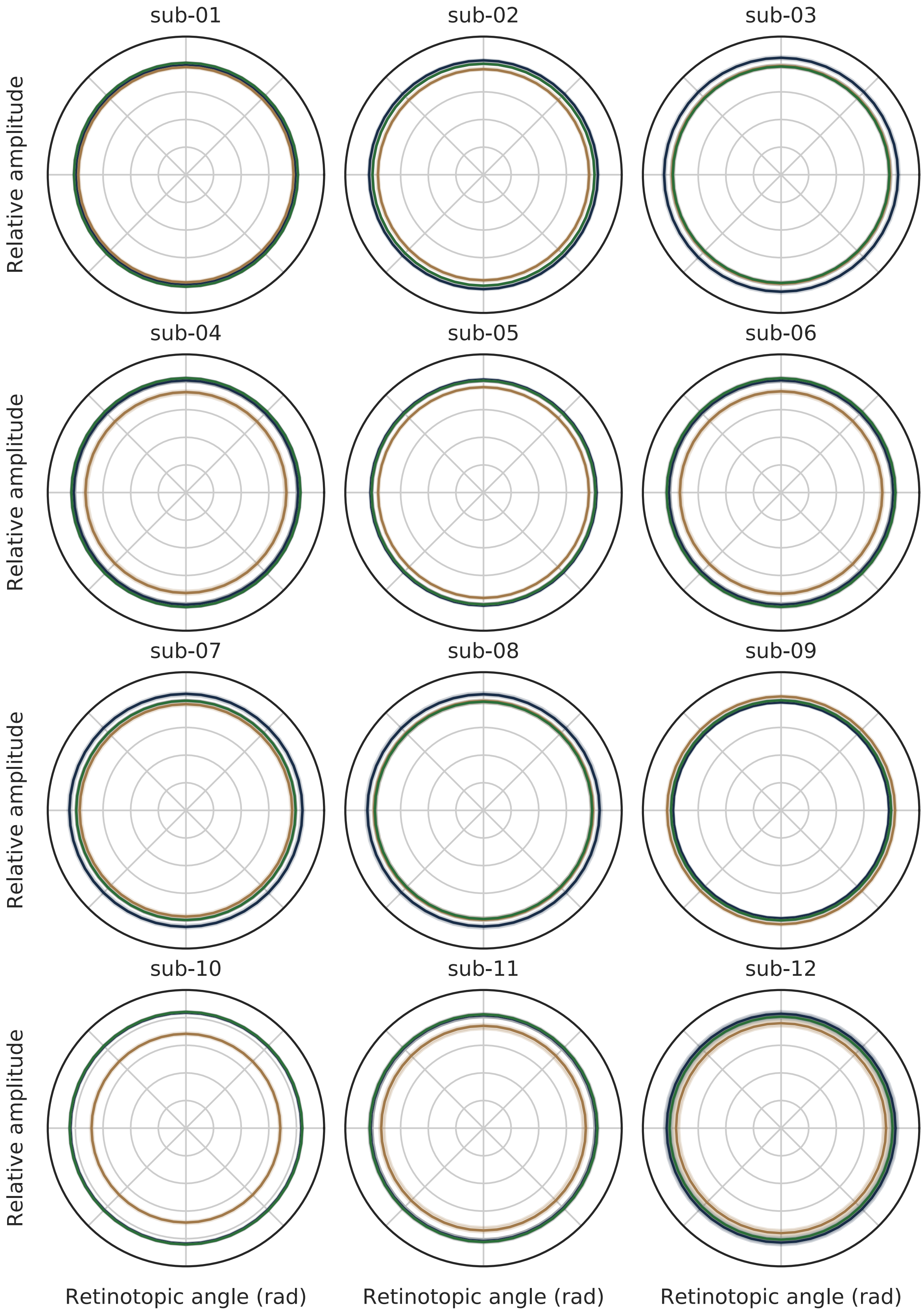

Figure S11: Individual subjects' relative gain as a function of retinotopic angle for absolute reference frame (as in bottom right panel of figure 10B). Lines show the median parameter and shaded regions cover the 68% confidence intervals obtained from bootstrapping across fMRI runs.
